# A pivot mutation impedes reverse evolution across an adaptive landscape for drug resistance in *Plasmodium vivax*

**DOI:** 10.1186/s12936-016-1090-3

**Published:** 2016-01-25

**Authors:** C. Brandon Ogbunugafor, Daniel Hartl

**Affiliations:** Department of Biology, University of Vermont, Burlington, VT USA; Vermont Complex Systems Center, The University of Vermont, Burlington, VT USA; Department of Organismic and Evolutionary Biology, Harvard University, Cambridge, MA USA

**Keywords:** *Plasmodium vivax*, Pyrimethamine resistance, Adaptive trajectories, Reverse evolution, Gene by environment interactions

## Abstract

**Background:**

The study of reverse evolution from resistant to susceptible phenotypes can reveal constraints on biological evolution, a topic for which evolutionary theory has relatively few general principles. The public health catastrophe of antimicrobial resistance in malaria has brought these constraints on evolution into a practical realm, with one proposed solution: withdrawing anti-malarial medication use in high resistance settings, built on the assumption that reverse evolution occurs readily enough that populations of pathogens may revert to their susceptible states. While past studies have suggested limits to reverse evolution, there have been few attempts to properly dissect its mechanistic constraints.

**Methods:**

Growth rates were determined from empirical data on the growth and resistance from a set of combinatorially complete set of mutants of a resistance protein (dihydrofolate reductase) in *Plasmodium vivax,* to construct reverse evolution trajectories. The fitness effects of individual mutations were calculated as a function of drug environment, revealing the magnitude of epistatic interactions between mutations and genetic backgrounds. Evolution across the landscape was simulated in two settings: starting from the population fixed for the quadruple mutant, and from a polymorphic population evenly distributed between double mutants.

**Results:**

A single mutation of large effect (S117N) serves as a pivot point for evolution to high resistance regions of the landscape. Through epistatic interactions with other mutations, this pivot creates an epistatic ratchet against reverse evolution towards the wild type ancestor, even in environments where the wild type is the most fit of all genotypes. This pivot mutation underlies the directional bias in evolution across the landscape, where evolution towards the ancestor is precluded across all examined drug concentrations from various starting points in the landscape.

**Conclusions:**

The presence of pivot mutations can dictate dynamics of evolution across adaptive landscape through epistatic interactions within a protein, leaving a population trapped on local fitness peaks in an adaptive landscape, unable to locate ancestral genotypes. This irreversibility suggests that the structure of an adaptive landscape for a resistance protein should be understood before considering resistance management strategies. This proposed mechanism for constraints on reverse evolution corroborates evidence from the field indicating that phenotypic reversal often occurs via compensatory mutation at sites independent of those associated with the forward evolution of resistance. Because of this, molecular methods that identify resistance patterns via single SNPs in resistance-associated markers might be missing signals for resistance and compensatory mutation throughout the genome. In these settings, whole genome sequencing efforts should be used to identify resistance patterns, and will likely reveal a more complicated genomic signature for resistance and susceptibility, especially in settings where anti-malarial medications have been used intermittently. Lastly, the findings suggest that, given their role in dictating the dynamics of evolution across the landscape, pivot mutations might serve as future targets for therapy.

**Electronic supplementary material:**

The online version of this article (doi:10.1186/s12936-016-1090-3) contains supplementary material, which is available to authorized users.

## Background

In recent years, experts have introduced several new perspectives on the management of drug resistance in malaria and other infectious diseases. These include criticisms of the aggressive use of therapeutic agents [1–3], the broader encouragement of more responsible use of antimicrobials [4–7] and the exploration of drug cycling strategies [8–13]. Drug stewardship programmes have been successful in several settings, and declines in drug resistance have been observed following changes in antibiotic use [14, 15]. There are other settings, however, where more careful use of antibiotics was not so effective, microbial populations remaining highly resistant even after removal of drug [16–19], an outcome with serious health and financial consequences. Pathogens might remain resistant to antimicrobials even after their removal for several reasons, among them compensatory mutations at other loci that counteract any fitness cost of drug resistance [18, 20, 21]. While compensatory mutations at other loci underlie many long-term fixation patterns in clinical infections, it is not fully understood why compensatory mutation is necessary, rather than the evolutionary undoing of mutations that ‘fixed’ in the process of forward resistance evolution.

The lack of a coherent understanding of reverse evolution is partly due to conceptual ambiguity: the term ‘reverse evolution’ is misleading, as it implies directionality in a process (Darwinian evolution) that is near-sighted and agnostic with regard to goal. This has spawned similarly dubious concepts, such as Dollo’s Law, asserting that evolution is intrinsically irreversible [22] because it would require two independent, low-probability events, occurring along the same pathway, but in opposite order [23]. Consequently, few studies have examined the molecular pathways through which reverse evolution across an antimicrobial resistance adaptive landscape is likely to occur. One such study of cefotaxime/pipericillin resistance in *Escherichia coli* highlighted that epistasis may wire ‘hidden randomness’ into adaptive landscapes that prevents reverse evolution [24]. A landmark study of reverse evolution in the vertebrate glucocorticoid receptor identified a combination of five mutations, labelled an ‘epistatic ratchet’, that precludes evolution towards the ancestral state [25]. Studies of this sort are even less frequent as they pertain to the problem of malaria drug resistance, which remains the cause of a global pandemic complicated by widespread resistance [26].

Approaches utilizing all possible combinations of a suite of mutations associated with resistance can help to resolve the likelihood of adaptive evolution occurring through certain pathways [27–31]. This study uses empirical data from a combinatorial analysis of *Plasmodium vivax* dihydrofolate reducatase (DHFR) mutants, evolutionary theory, and individual-based simulations to uncover factors that affect the likelihood of reverse evolution across pyrimethamine (PYR) concentrations. In doing so, it proposes a method for determining whether reverse evolution will occur across an adaptive landscape. By measuring the fitness effects of individual mutations, the study uncovers the existence of a mutational pivot with potentiated genotype-by-environment (G × E) effects that may direct evolution towards or constrain evolution from areas of the landscape with high resistance or fitness. In addition, these mutations attract interactions with other mutation sites, creating an epistatic ratchet, limiting reverse evolution across a landscape. Lastly, the study discusses the implications of these findings for evolutionary theory, molecular epidemiology and in two clinically relevant contexts: (1) the use of existing drugs for resistance management in malaria, and, (2) the rational design of drugs that might target certain amino acid residues of a resistance determinant.

## Methods

### System of study and growth rates

The study modelled empirical growth and resistance (IC_50_) data developed in a prior study [32] in strains of transgenic *Saccharomyces cerevisiae* carrying *P. vivax* DHFR containing a set of four mutations orthologous to the resistance mutations found in *Plasmodium falciparum* [30], in all combinations, several of which have been isolated from field settings [33–46]. The combinatorial approach is an effective way to create empirical adaptive landscapes for final phenotypes when all intermediate genotypes can be reconstructed in the protein of interest, often in a transgenic setting (*Saccharomyces cerevisiae* in this case). Because of this, this approach is not meant to be a literal analogue for drug treatment, but does effectively test important properties of protein evolution. Results derived from prior studies of this kind have recapitulated findings from the field [29, 32], reaffirming that this approach has utility in understanding the evolution of drug resistance.

**Fig. 1:**
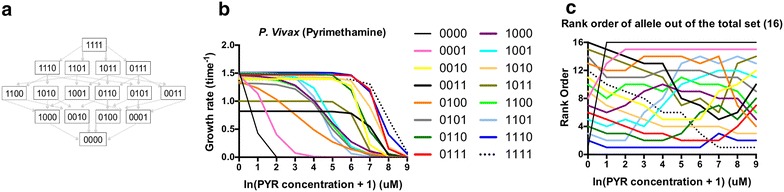
Alleles composing the *Plasmodium vivax* adaptive landscape for drug resistance in this study. **a** Schematic of the possible pathways between the most resistant allele (1111) and most susceptible (0000). **b** Growth rates of alleles in the landscape of *P. vivax* as observed in Jiang et al. [32] **c** Rank order curves for *P. vivax* in pyrimethamine. The *y*-axis depicts the rank order of alleles at a given drug concentration. The *x*-axis is in terms ln(concentration of PYR + 1) of the pyrimethamine drug concentration in μM. Note how regularly the lines intersect across drug concentrations. This indicates G × E interactions, which alters the structure of the landscape

This study used bit-string notation, with 0 corresponding to the presence of the ancestral state mutation, and 1 a replacement of a mutation observed to confer varying levels of fitness in the presence of drug concentrations. The individual amino acid sites are N50I (1***), S58R (*1**), S117N (**1*), and I173L (***1). A diagram of the possible evolutionary trajectories from the state 1111 to 0000 is shown in Fig. 1a.

A logistic growth equation was used to model growth rates across a range of concentrations (Fig. 1b). The growth equation, as described in Jiang et al. [32] is as follows:

1 $$ g\left( x \right) = \frac{{g_{drugless} }}{{1 + e^{{\frac{{{\text{IC}}_{50} - x}}{c}}} }} $$

where *g*_*drugless*_ is the growth rate in the presence of no drug, the IC_50_ value in μM and the *c* a constant that determines the slope of the curve. The estimated growth rates are robust with regard to estimation error, as the standard errors of the estimated IC_50_ parameters are quite low, generally less than 10 % of the mean (Additional file 1). The growth rates in the study are determined across a broad range (Fig. 1b, Additional file 2) which includes those concentrations of PYR observed in the blood of persons treated with PYR [47–51]. The study used these growth rates to determine the accessibility of pathways, as in prior studies. In order to identify an accessible pathway, the rank orders between alleles must decrease from step-to-step, indicating that a mutation is moving to a higher fitness portion of the landscape (a ranking of 1 meaning the most fit allele in the landscape). Figure 1c demonstrates how the rank order of fitnesses changes as a function of drug concentration (values in Additional file 3). For the purposes of this study, one should note how often the lines cross one another at different drug concentrations. This indicates the presence of gene by environment (G × E) interactions that alter the structure of the adaptive landscape and create different evolutionary dynamics in different drug environments.

### Fitness effect of mutations

To estimate the interaction between the effect of mutation and drug concentration (G × E interaction), the effect of individual mutations across drug concentrations was calculated [52]. For the *P. vivax* DHFR landscape, each mutant site has eight possible genetic backgrounds to which it could be added. To calculate the effect of a mutation, take the difference between the fitness (*W*) of an allele *j* and the one-step neighbour carrying mutation ε, where ε corresponds to mutations: N50I (1***), S58R (*1**), S117N (**1*), and I173L (***1):

2 $$ \Delta W_{\varepsilon } = W_{j} - W_{{j_{\varepsilon } }} $$

This was calculated for each of the four mutations across a range of drug concentrations (between 0 and approximately 8000 μM). After calculating the fitness effect of mutations across drug concentrations, the average fitness of all whole alleles carrying each of the four mutations (1***, 1***, **1*, ***1) was measured and compared using ANOVA to determine any significant differences between mutant classes (Additional file 4).

### Measuring epistasis

Embedded in Fig. 3a is epistasis, or the “surprise at the phenotype when mutations are combined, given the constituent mutations’ individual effects” [52]. Epistasis was measured by calculating the standard deviation of the total fitness effects for a mutation at a given concentration. These values were plotted in Additional file 5.

### Simulations of evolution

Having identified the S117N mutation as having the greatest G x E effect, computer simulations were used to test whether these (or other) effects constrain evolution in certain directions. SimuPop was used as the simulation convention, an individual-based, Wright–Fisher, forward-time simulation model [53] similar to that described by Jiang et al. [32]. In this model, generations are discrete (non-overlapping), with an effective population size of 10,000. Mutation rates were determined by the relative rate matrix for *P. vivax* computed from data in Neafsey et al. [54]. For the purposes of converting the relative substitution rate matrix to a more realistic per-generation rate matrix, mutation rates were divided by 10^3^ and then converted into amino acid substitution rates using the sum of substitution rates of nucleotides responsible for drug resistance in *P. vivax* DHFR.

The mutation rates were also scaled by a factor of 1000 to allow simulations with fewer individuals. Scaling involves dividing the population size by a scaling factor, *m*, and then multiplying the mutation rates by that same factor:

3 $$ N_{e} \cdot \mu = \frac{{N_{e} }}{m}\left( {\mu \cdot m} \right) $$

The simulations were designed to simulate the dynamics of reverse evolution in two population genetic scenarios:

1. A population fixed for the most resistant (1111) allele for 1000 generations across a range of drug concentrations (~3000, ~400, ~55, ~7 μM). This allows one to observe the general dynamics of reverse evolution, and test whether reversion towards the wild type (0000) ever occurs. The most obvious prediction would be that at the extremely high PYR concentration (~3000 μM), the population should remain trapped on the 1111 allele, as it is the most resistant allele in the set and has the highest growth rate at the highest concentration (Fig. 1b, c, Additional file 3).
2. A population composed equally of all six double mutants (1100, 1010, 1001, 0011, 0101, 0110), evolving in the absence of drug. Because the double mutants are in the centre of the landscape (in terms of Hamming distance between 0000 and 1111), simulations with them as a starting point would uncover any intrinsic landscape bias towards forward or reverse evolution.

## Results

### The structure of reverse evolution trajectories

**Fig. 2:**
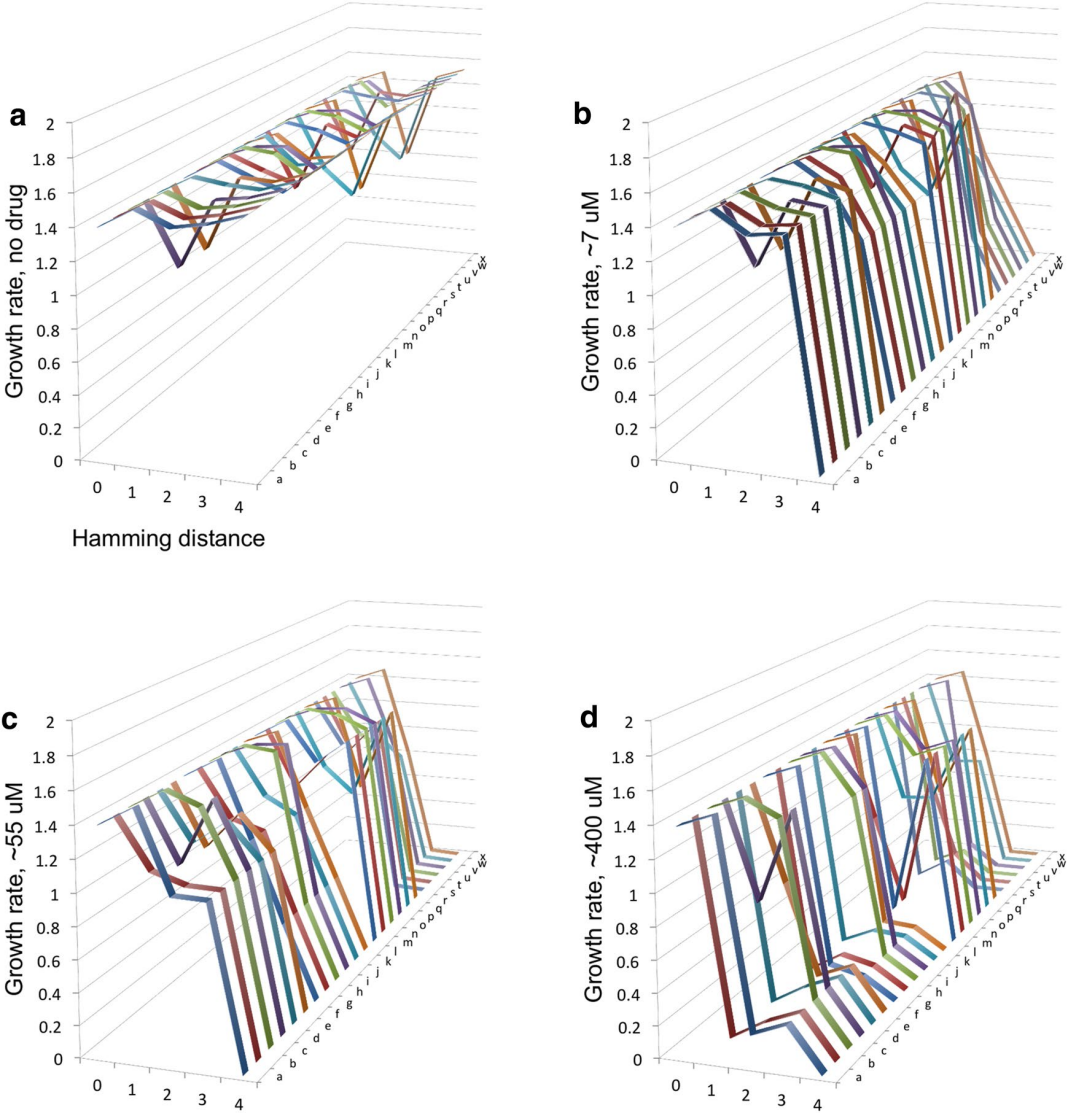
The structure of reverse evolution trajectories in *Plasmodium vivax*. Twenty-four adaptive landscapes for *P. vivax* DHFR across several drug concentrations, organized into individual trajectories. The *y*-axis is growth rate. The *x*-axis denotes hamming distance from the original, which is the quadruple mutant (1111) in this study (0 = quadruple mutant,* 1* triple mutant,* 2* double mutant,* 3* singe mutant,* 4* ancestral allele), and the *z*-axis corresponds to the 24 different possible pathways between the most resistant allele (1111) and the ancestral allele (0000). Additional file 6 identifies the individual pathways *a*–*x*. Growth rates are in units of time^−1^. Concentrations: **a** no drug, **b** ~7 μM, **c** ~55 μM, **d** ~400 μM

Using fitness values for *P. vivax* based on Eq. 1, three-dimensional representations of all possible trajectories were constructed for each drug across several drug concentrations (Fig. 2). In particular, one should highlight the structure of the reverse evolutionary trajectories at the two lower drug concentrations (no drug and ~7 μM, Fig. 2a, b), as the wild-type ancestor (0000) has a relatively high fitness in both (Fig. 1). Note the presence of fitness valleys in all trajectories between 1111 and 0000, even at low concentrations, indicating that 0000 is inaccessible through mutation-selection balance alone. This characteristic of the trajectories is further examined in other parts of this study. Keep in mind that the quadruple mutant (1111) might not exist in nature for *P. vivax* DHFR. This means that this exact scenario might not reflect how reversal occurs in nature, but still changes little about the purpose or relevance of the study: to diagnose features of the adaptive landscape that explain why reverse evolution might be difficult, rather than explain any single finding in any particular ecological context. In order to do so, evolution was modelled from one extreme of the landscape (1111) to the other (0000), all towards a conceptual and mechanistic understanding of the constraints on reverse evolution, suggesting forces at play in wild populations of malaria parasite.

### Analysis of the fitness effects of mutations across drug concentrations reveals a single mutation of uncommonly large positive effect

**Fig. 3:**
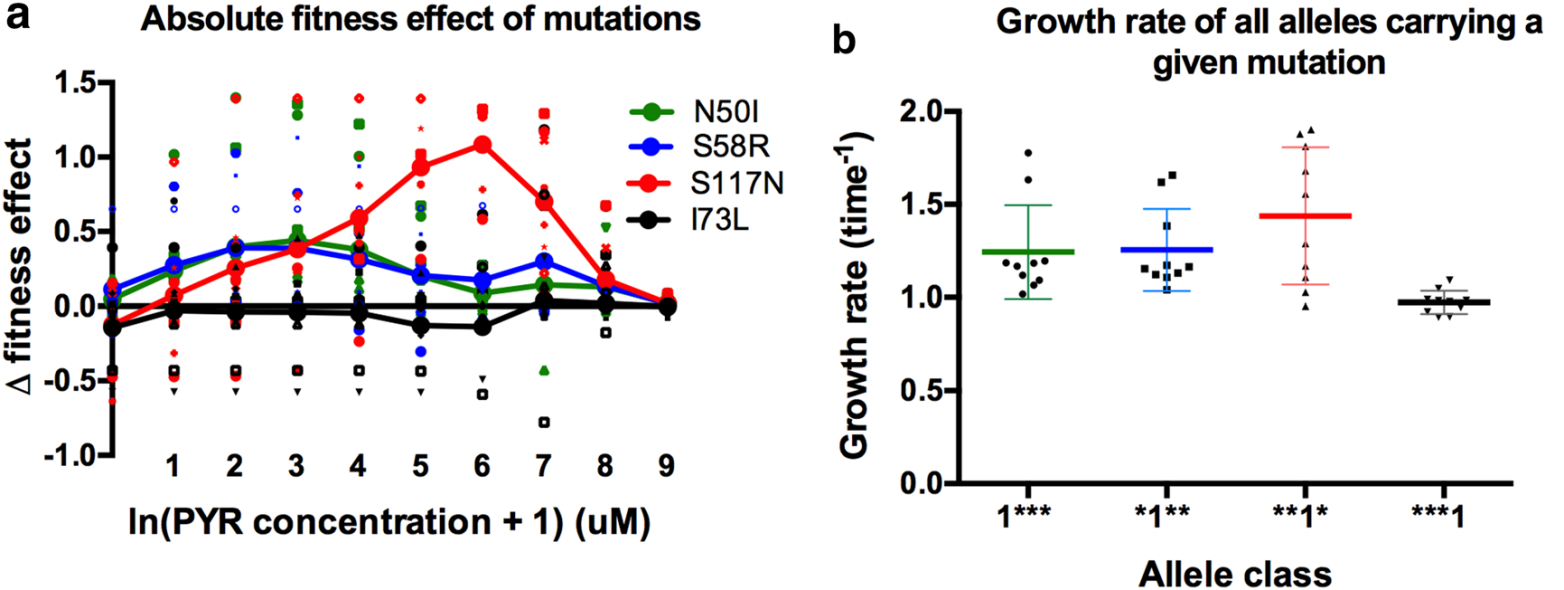
A single mutation potentiator of G × E effects, S117N, has uncommonly high fitness effects across a range of drug concentrations, and creates alleles that are of higher fitness. **a** *Each colour* represents the Δ fitness effect of a one of the four mutations, the difference between a genotype with and without a mutation. Each mutation has 8 possible genetic backgrounds. *Small* (unconnected) *symbols* are representative of Δ fitness effect measures for individual mutation effects in a particular genetic background. *Large symbols*, connected by lines, represent the average Δ fitness effect of a mutation over all 8 genetic backgrounds. The third site (S117N) mutation has a strong effect, creating high fitness alleles that foster forward evolution and inhibit reverse evolution. The *x*-axis is in units ln(concentration of PYR + 1) μM.** b** Whole alleles carrying the G × E pivot mutation (**1*) have significantly higher growth rates than the other alleles (averaged across drug concentrations). This graph differs from **a** because this does not depict fitness effects of single mutations, but rather, the total average fitness of whole alleles carrying the specified mutation

The fitness effect of individual mutations across drug concentrations was then measured. Figure 3a displays both the average effects (solid lines) and individual effect points (scattered points). Here, one can see that the third site mutation, S117N (**1*) has a strongly positive fitness effect across environments (*P* = 2.22 × 10^−8^, Additional file 4). To observe how this mutation contributes to the fitness of alleles composing the landscape, the average growth rate of all alleles that carry each individual mutation was then calculated (Fig. 3b). This analysis reveals that alleles containing the S117N mutation have significantly higher growth rates across environments than alleles carrying the other mutations (*P* = 0.025; F = 5.7; df = 3, 36) (Fig. 3b).

### Simulations of evolution

**Fig. 4:**
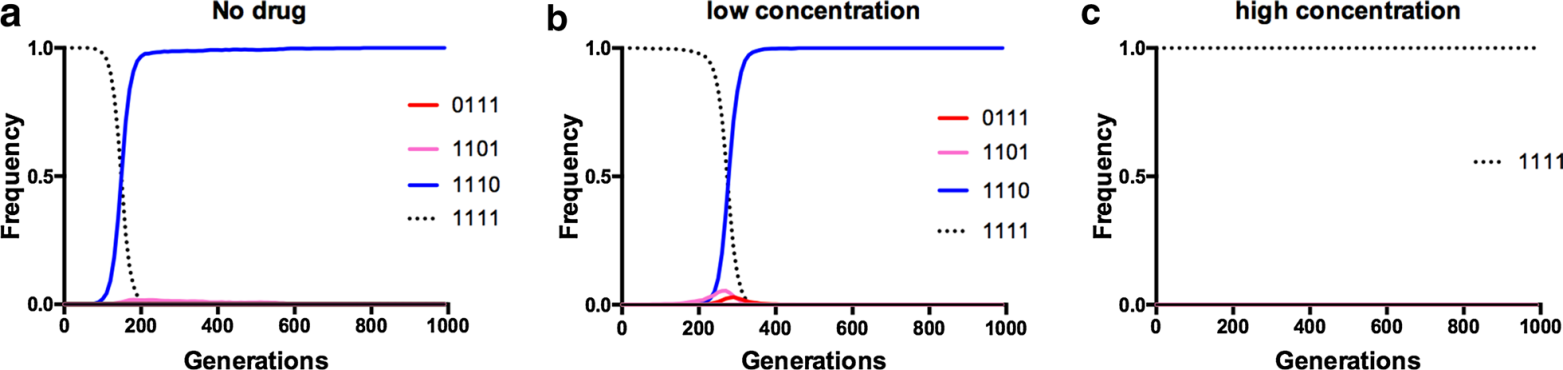
Starting from the quadruple mutant (1111), reverse evolution towards the ancestor (0000) is impeded across concentrations. These are illustrative examples of the most preferred pathways for evolution at each of the simulated pyrimethamine environments, starting with the 1111 quadruple mutant. *Panels* correspond to several simulation scenarios: **a** no drug, **b** low drug (~7.0 μM), **c** high drug (~3000 μM). In each case, one observes no accessible trajectories in the fitness landscape leading to the ancestral allele (0000), not even in the no-drug environment where the ancestor is the most fit

**Fig. 5:**
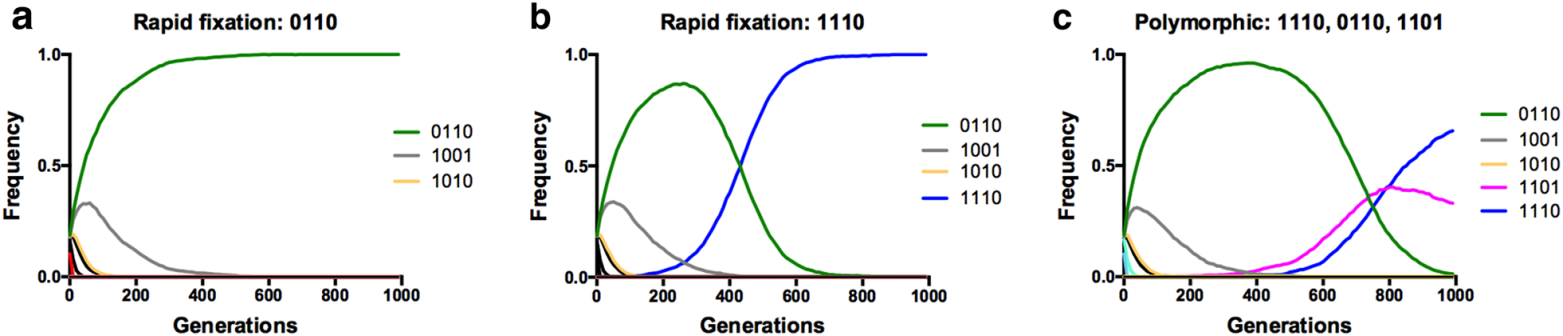
Evolution starting from the ‘centre’ of the landscape (double mutants) cannot cross the single mutant valley en route to the wild type ancestor (0000). These are illustrative examples of individual simulations of evolution without drug when the starting population is composed equally of the double mutants: 1100, 1010, 1001, 0011, 0101, and 0110. Broadly speaking, one can observe three different outcomes after 1000 generations: **a** fixation of the third-site (**1*) in the double mutant (0110), **b** a third-site-carrying triple mutant (1110) or **c** a “polymorphic” population with 0110, 1101 and 1110 all present in frequency space after 1000 generations (but headed towards an eventual fixation of 1110). No simulations starting from the centre moved in the *reverse direction* toward the ancestral allele (0000)

Next, evolution was simulated across the adaptive landscape to test whether S117N plays a key role in impeding reversal in two settings: starting from (1) a population composed of the most resistant allele (1111), and, (2) from the centre of the landscape with a population divided equally between each of the six double mutants (see “Methods”). Figures 4 and 5 depict illustrative dynamics of evolution in these simulations, and Table 1 contains a more detailed summary of all simulation runs.

**Table 1:** Summary of simulations of evolution in two schemes

(a) Reverse evolution simulation summary		
Starting conditions (drug and allele)	Outcome	Fraction
~3000 μM PYR (peak mutant = 1111)		
1111	1111	0.97
1111	1111 → 0111	0.3
~400 μM PYR (peak mutant = 1110)		
1111	1111 → 1110	0.93
1111	1111 → 0111	0.06
~55 μM PYR (peak mutant = 1110)		
1111	1111 → 1110	0.96
1111	1111 → 0111 → 0110	0.04
~7 μM PYR (peak mutant = 1110)		
1111	1111 → 1110	0.97
1111	1111 → 1101	0.03
No drug (peak mutant = 0000)		
1111	1111 → 1110	0.91
1111	1111 → 1101	0.08

(b) Double mutants, no drug simulation summary	
Outcome	Fraction
Equally distributed: 1100, 1001, 1010, 0011, 0101, 1110	
Polymorphic → 1110	0.54
Polymorphic → 0110	0.27
Polymorphic → 1101	0.19

(a) Starting from a population fixed for 1111, across drug concentrations and (b) starting from the centre of the landscape, a population composed equally of the six double mutants. Note that this summary includes several drug concentrations not visualized in Figs. 4 and 5. For (a) which allele in the landscape is the absolute peak at that drug concentration is also highlighted (in parentheses). In a smooth landscape, the landscape should be able to locate the absolute peak. Note that in the no drug environment, the population remains trapped on triple mutant local peaks (1110 and 1101), and unable to locate the 0000 absolute peak

Simulations demonstrate that populations fixed for the 1111 allele do not undergo reverse evolution to the ancestral allele at any drug concentration (including the drugless environment) even after a thousand generations, but are trapped at the 1110 triple mutant fitness peak at most concentrations, with a small fraction of simulations leading to the 0111 triple mutant (Fig. 4; Table 1). This finding reflects the fact that the 1110 triple mutant has a high growth rate even in the drugless environment, superior to all of its double- and single-mutant neighbours. Although the 0110, 1110 and 0111 alleles (all of which contain the S117N mutation) have a growth rate lower than the ancestor (0000) in the no-drug environment, evolving populations are unable to cross the single-mutant (1000, 0100, 0010, 0001) valley necessary to reach the ancestral genotype, precluding reverse evolution (Fig. 5). This is because the combination of the S117N mutation and the second-site mutation, S58R (*1**) has properties of an epistatic ratchet [25] that restricts reverse evolution: it is able to reproduce well enough at both higher drug concentrations and in the drugless environment (Fig. 1b) to limit crossing the single mutation (0010 and 0100 in this case) valley necessary to reach the 0000 absolute fitness peak in the drugless environment.

## Conclusions

While irreversibility across an adaptive landscape for antimicrobial resistance has been observed in many pathogen types, this question has been relatively unexplored in malarial parasites and in particular, as it pertains to a mechanism underlying this constraint. In the case of malaria, several past studies from the field, involving both chloroquine and pyrimethamine, support the assertion that reverse evolution is improbable: in one instance, a population of *P. falciparum* resistant to chloroquine reverted to wild type only after replacement with a migrant population composed of ancestral susceptible genotypes (rather than through *de novo* mutation and selection) [55]. In another, a population of *P. falciparum* resistant to pyrimethamine compensated through copy number variation in GTP cyclohydrolase in lieu of reversing the mutations already fixed in DHFR [56].

Although the study focused on *P. vivax* DHFR, it provides a conceptual basis for irreversibility in other resistance proteins. The findings from the field, in combination with these results, imply that modern whole-genome sequencing efforts will reveal a more complex genomic signature of resistance and reversal in settings where antimicrobial use has waxed and waned. This has implications for the practice of molecular epidemiology: while sequencing selected SNPs in resistance determinants might be sufficient for identifying resistance alleles in settings where populations of pathogen are ‘forward’ evolving resistance, the genomic picture is likely more complicated in reverse. The results, corroborated by findings from the field, suggest that re-evolution of the susceptible phenotype (without the growth defects of the more resistant phenotypes) is likely to occur either through the introduction of susceptible migrant genotypes from elsewhere or compensatory mutations at sites other than the ones originally arising during resistance evolution [57, 58].

This study dissected barriers to reverse evolution from the most resistant genotype (1111) toward the most susceptible (0000) across an adaptive landscape for drug resistance mediated by DHFR in *P. vivax*. Among four amino acid replacements resulting in pyrimethamine resistance, a single site, S117N (**1*) had a strong effect on the fitness of alleles in the landscape across a breadth of drug concentrations. At high drug concentrations, double and triple mutants containing the S117N mutation, and in particular those in combination with the second site mutation S58R (0110 and 1110, for example) have a reproductive advantage across most drug environments. More specifically, while these higher order alleles have lower fitness than the 0000 ancestral allele in the absence of drug, they have substantially higher fitness than the single mutant neighbours that separate the higher-order mutants carrying the S117N (**1*) mutation (1000, 0100, 0010, 0001), which explains the low likelihood of reverse evolution across this drug resistance adaptive landscape.

Simulations of evolution across the landscape demonstrate the consequences of genotype-by-environment interactions involving S117N: whereas past studies have shown that forward evolution from the 0000 ancestor to an absolute fitness peak occurs readily at drug concentrations greater than 0 [32], evolution starting from the population fixed for the 1111 quadruple mutant becomes trapped at the 1110 triple mutant local fitness peak, even in the drugless environment (Fig. 4). Even more, when the landscape starts with a population distributed equally between the double mutants (the centre of the landscape; 0011, 0101, 1001, 1010, 0110, 1100), the evolutionary dynamics are still driven by the S117N site (**1*), usually resulting in fixation of the 1110 triple mutant (Fig. 5; Table 1). In this sense, the S117N mutation serves as a pivot point for mutation: its arrival provides a bridge to high fitness areas of the landscape that are trapped onto local peaks through their interaction with other mutations, unable to move to other areas of the landscape.

These findings support the existence of epistatic ratchets that inhibit reverse evolution towards ancestral states, such as that observed in the evolution of the vertebrate glucocorticoid receptor [25]. While the pivotal S117N mutation creates a ratchet through epistatic interactions, its average effect alone (across all genetic backgrounds and across environments) is larger than that of the other sites (1***, * 1**, ***1), indicating that its fitness effects are not limited to singular genetic backgrounds or certain environments. In this sense, S117N opens evolutionary ‘forks in the road’ towards higher mutation regions of the landscape (double mutants, triple mutants and the quadruple mutant), serving as the starting material for the epistatic ratchets that ultimately prevent reverse evolution.

Other than the implications for molecular epidemiology discussed above, these findings are most relevant to debates surrounding best practices for antimicrobial resistance management. The notion that ceasing use of antimicrobials is a viable strategy for decreasing resistance is based, in part, on the assumption that reverse evolution can occur across a landscape because of the fitness cost of resistance. The results suggest that such strategies may not be generally valid, and they should be tailored to the nature of the actual adaptive landscape and its G × E interactions affecting the accessible trajectories towards resistance and susceptibility. Please note that these comments apply to stepwise reverse evolution in a situation where a derived resistance allele (1111 in this manuscript) is fixed. Alternatively, if a population retains the ancestral allele at low frequency, it can increase in frequency in the absence of drug. This latter scenario is not stepwise evolution, however, but canonical selection on standing genetic variation, a different population genetic context than the one simulated in this study.

Lastly, and most provocatively, the identification of the S117N pivot mutation suggests a possible strategy for identifying targets for chemotherapeutic intervention: if a single mutation is a pivot point to large fitness effects (as found in this study), it might be an ideal drug target. Compounds that perturb the interaction between these pivot residues and others might have a destabilizing effect on protein structure or function, and diminish the evolutionary potential of alleles carrying the resistance determinant. Because this strategy would target the ability of a protein to reach high fitness areas of adaptive landscapes, it would constitute a strategy directed against the evolvability of the pathogen, an unexplored avenue for the treatment of microbial pathogens.

## Additional files

10.1186/s12936-016-1090-3 Values and standard errors for the empirical derived parameters used to model growth rates: Drugless growth rates, IC50 values.
10.1186/s12936-016-1090-3 Standardized simulated growth rates as depicted in Fig. 1b.
10.1186/s12936-016-1090-3 The rank order of alleles as depicted in Fig. 1.
10.1186/s12936-016-1090-3 ANOVA: Interaction between mutation effect and drug concentration for Plasmodium vivax in the presence of pyrimethamine. All values for dfnum.,denom. = 9, 70.
10.1186/s12936-016-1090-3 Standard deviation of the absolute fitness effects of a mutation (epistasis). Epistasis can be measured any number of ways, however, the standard deviation provides a proxy: it measures the dispersion of G × G effects for a given mutation at a given drug.
10.1186/s12936-016-1090-3 Discrete pathways with letter corresponding to the paths in Fig. 2.
