# Supplementary material for: A pivot mutation impedes reverse evolution across an adaptive landscape for drug resistance in Plasmodium vivax
Source: Malar J. 2016 Jan 25;15:40. doi: 10.1186/s12936-016-1090-3 (PMC4727274; doi:10.1186/s12936-016-1090-3)
Supplement: Supplementary file 1 — 10.1186/s12936-016-1090-3 Values and standard errors for the empirical derived parameters used to model growth rates: Drugless growth rates, IC50 values. [file 12936_2016_1090_MOESM1_ESM.docx]

Additional File 1

|  | Drugless Growth | S.E. | Log (IC_50_) Pyrimethamine | S.E. |
| --- | --- | --- | --- | --- |
| 0000 | 1.398 | 0.0535 | -6.286 | 0.053 |
| 0001 | 1.275 | 0.0131 | -5.812 | 0.013 |
| 0010 | 1.227 | 0.0195 | -4.239 | 0.014 |
| 0100 | 1.370 | 0.0287 | -6.046 | 0.035 |
| 0101 | 1.375 | 0.0164 | -5.774 | 0.019 |
| 0110 | 1.397 | 0.0268 | -3.732 | 0.025 |
| 0111 | 1.219 | 0.0737 | -3.55 | 0.033 |
| 1000 | 1.119 | 0.0349 | -5.724 | 0.029 |
| 1001 | 1.184 | 0.0595 | -5.491 | 0.029 |
| 1010 | 1.306 | 0.0336 | -4.015 | 0.017 |
| 1011 | 1.000 | 0.0814 | -4.6 | 0.033 |
| 1100 | 1.273 | 0.0509 | -5.773 | 0.028 |
| 1101 | 1.282 | 0.0444 | -5.624 | 0.034 |
| 1110 | 1.450 | 0.0159 | -3.587 | 0.116 |
| 1111 | 1.250 | 0.0457 | -3.3 | 0.033 |

**Table S1.** Values and standard errors for the empirical derived parameters used to model growth rates: Drugless growth rates, IC_50_ values.
