# Supplementary material for: A pivot mutation impedes reverse evolution across an adaptive landscape for drug resistance in Plasmodium vivax
Source: Malar J. 2016 Jan 25;15:40. doi: 10.1186/s12936-016-1090-3 (PMC4727274; doi:10.1186/s12936-016-1090-3)
Supplement: Supplementary file 2 — 10.1186/s12936-016-1090-3 Standardized simulated growth rates as depicted in Fig. 1b. [file 12936_2016_1090_MOESM2_ESM.docx]

Additional File 2

|  | Natural log (pyrimethamine + 1) (uM) | | | | | | | | | |
| --- | --- | --- | --- | --- | --- | --- | --- | --- | --- | --- |
|  | **0** | **1** | **2** | **3** | **4** | **5** | **6** | **7** | **8** | **9** |
| 0000 | 1.53 | 0.43 | 0.00 | 0.00 | 0.00 | 0.00 | 0.00 | 0.00 | 0.00 | 0.00 |
| 0001 | 1.46 | 1.13 | 0.40 | 0.07 | 0.01 | 0.00 | 0.00 | 0.00 | 0.00 | 0.00 |
| 0010 | 1.40 | 1.40 | 1.40 | 1.40 | 1.40 | 1.39 | 1.27 | 0.22 | 0.01 | 0.00 |
| 0011 | 0.82 | 0.82 | 0.82 | 0.82 | 0.82 | 0.81 | 0.78 | 0.55 | 0.04 | 0.00 |
| 0100 | 1.35 | 1.23 | 1.03 | 0.76 | 0.49 | 0.27 | 0.14 | 0.06 | 0.03 | 0.01 |
| 0101 | 1.32 | 1.31 | 1.29 | 1.22 | 0.96 | 0.50 | 0.16 | 0.04 | 0.01 | 0.00 |
| 0110 | 1.49 | 1.49 | 1.49 | 1.49 | 1.49 | 1.47 | 1.19 | 0.46 | 0.06 | 0.00 |
| 0111 | 1.47 | 1.47 | 1.47 | 1.47 | 1.47 | 1.47 | 1.46 | 1.21 | 0.15 | 0.00 |
| 1000 | 1.46 | 1.45 | 1.40 | 1.28 | 1.01 | 0.60 | 0.27 | 0.10 | 0.04 | 0.01 |
| 1001 | 1.47 | 1.47 | 1.47 | 1.43 | 1.23 | 0.67 | 0.19 | 0.04 | 0.01 | 0.00 |
| 1010 | 1.43 | 1.43 | 1.43 | 1.43 | 1.43 | 1.42 | 1.36 | 0.90 | 0.18 | 0.02 |
| 1011 | 1.00 | 1.00 | 1.00 | 1.00 | 1.00 | 0.99 | **0.77** | 0.12 | 0.00 | 0.00 |
| 1100 | 1.42 | 1.41 | 1.38 | 1.27 | 1.01 | 0.57 | 0.19 | 0.05 | 0.02 | 0.00 |
| 1101 | 1.51 | 1.50 | 1.49 | 1.43 | 1.08 | 0.37 | 0.06 | 0.01 | 0.00 | 0.00 |
| 1110 | 1.51 | 1.51 | 1.51 | 1.51 | 1.51 | 1.51 | 1.46 | 1.17 | 0.40 | 0.06 |
| 1111 | 1.39 | 1.39 | 1.39 | 1.39 | 1.39 | 1.39 | 1.39 | 1.30 | 0.68 | 0.08 |

**Table S2.** Standardized simulated growth rates as depicted in Figure 1B.
