# Supplementary material for: A pivot mutation impedes reverse evolution across an adaptive landscape for drug resistance in Plasmodium vivax
Source: Malar J. 2016 Jan 25;15:40. doi: 10.1186/s12936-016-1090-3 (PMC4727274; doi:10.1186/s12936-016-1090-3)
Supplement: Supplementary file 3 — 10.1186/s12936-016-1090-3 The rank order of alleles as depicted in Fig. 1. [file 12936_2016_1090_MOESM3_ESM.docx]

Additional File 3

|  | Natural log (pyrimethamine + 1) (uM) | | | | | | | | | |
| --- | --- | --- | --- | --- | --- | --- | --- | --- | --- | --- |
|  | **0** | **1** | **2** | **3** | **4** | **5** | **6** | **7** | **8** | **9** |
| 0000 | 1 | 16 | 16 | 16 | 16 | 16 | 16 | 16 | 16 | 16 |
| 0001 | 8 | 13 | 15 | 15 | 15 | 15 | 15 | 15 | 15 | 15 |
| 0010 | 11 | 9 | 8 | 7 | 5 | 5 | 5 | 9 | 10 | 12 |
| 0011 | 16 | 15 | 14 | 13 | 13 | 8 | 7 | 5 | 6 | 10 |
| 0100 | 13 | 12 | 12 | 14 | 14 | 14 | 13 | 14 | 7 | 4 |
| 0101 | 14 | 11 | 11 | 11 | 12 | 12 | 12 | 11 | 11 | 9 |
| 0110 | 4 | 3 | 3 | 2 | 2 | 3 | 6 | 7 | 5 | 8 |
| 0111 | 6 | 5 | 4 | 3 | 3 | 2 | 2 | 2 | 4 | 7 |
| 1000 | 7 | 6 | 7 | 9 | 10 | 9 | 9 | 8 | 8 | 5 |
| 1001 | 5 | 4 | 5 | 4 | 7 | 10 | 11 | 12 | 12 | 11 |
| 1010 | 9 | 7 | 6 | 5 | 4 | 4 | 4 | 4 | 3 | 3 |
| 1011 | 15 | 14 | 13 | 12 | 11 | 7 | 8 | 6 | 13 | 14 |
| 1100 | 10 | 8 | 10 | 10 | 9 | 11 | 10 | 10 | 9 | 6 |
| 1101 | 3 | 2 | 2 | 6 | 8 | 13 | 14 | 13 | 14 | 13 |
| 1110 | 2 | 1 | 1 | 1 | 1 | 1 | 1 | 3 | 2 | 2 |
| 1111 | 12 | 10 | 9 | 8 | 6 | 6 | 3 | 1 | 1 | 1 |

**Table S3.** The rank order of alleles as depicted in Figure 1
