# Supplementary material for: A pivot mutation impedes reverse evolution across an adaptive landscape for drug resistance in Plasmodium vivax
Source: Malar J. 2016 Jan 25;15:40. doi: 10.1186/s12936-016-1090-3 (PMC4727274; doi:10.1186/s12936-016-1090-3)
Supplement: Supplementary file 4 — 10.1186/s12936-016-1090-3 ANOVA: Interaction between mutation effect and drug concentration for Plasmodium vivax in the presence of pyrimethamine. All values for dfnum.,denom. = 9, 70. [file 12936_2016_1090_MOESM4_ESM.docx]

Additional File 4

**ANOVA**

Effect *F P*

Absolute effect of 1*** X environment 1.40 0.19

Absolute effect of *****1****** X environment 1.11 0.36

Absolute effect of **1* X environment 8.40 2.2 X 10^-8^

Absolute effect of ***1 X environment 0.37 0.94

**Table S4. ANOVA**: Interaction between mutation effect and drug concentration for *Plasmodium vivax* in the presence of pyrimethamine. All values for df _num., denom._ = 9, 70
