# Supplementary material for: A pivot mutation impedes reverse evolution across an adaptive landscape for drug resistance in Plasmodium vivax
Source: Malar J. 2016 Jan 25;15:40. doi: 10.1186/s12936-016-1090-3 (PMC4727274; doi:10.1186/s12936-016-1090-3)
Supplement: Supplementary file 5 — 10.1186/s12936-016-1090-3 Standard deviation of the absolute fitness effects of a mutation (epistasis). Epistasis can be measured any number of ways, however, the standard deviation provides a proxy: it measures the dispersion of G × G effects for a given mutation at a given drug. [file 12936_2016_1090_MOESM5_ESM.docx]

Additional File 5

**Figure S1. Standard deviation of the absolute fitness effects of a mutation (epistasis).** Epistasis can be measured any number of ways, however, the standard deviation provides a proxy: it measures the dispersion of G x G effects for a given mutation at a given drug.
