# Supplementary material for: A pivot mutation impedes reverse evolution across an adaptive landscape for drug resistance in Plasmodium vivax
Source: Malar J. 2016 Jan 25;15:40. doi: 10.1186/s12936-016-1090-3 (PMC4727274; doi:10.1186/s12936-016-1090-3)
Supplement: Supplementary file 6 — 10.1186/s12936-016-1090-3 Discrete pathways with letter corresponding to the paths in Fig. 2. [file 12936_2016_1090_MOESM6_ESM.docx]

Additional File 6

| Pathways as depicted in Figure 2 (main text) | |
| --- | --- |
| a | 0000-1000-1100-1110-1111 |
| b | 0000-1000-1100-1101-1111 |
| c | 0000-1000-1010-1110-1111 |
| d | 0000-1000-1010-1011-1111 |
| e | 0000-1000-1001-1101-1111 |
| f | 0000-1000-1001-1011-1111 |
| g | 0000-0100-1100-1110-1111 |
| h | 0000-0100-1100-1101-1111 |
| i | 0000-0100-0110-1110-1111 |
| j | 0000-0100-0110-0111-1111 |
| k | 0000-0100-0101-1101-1111 |
| l | 0000-0100-0101-0111-1111 |
| m | 0000-0010-1010-1110-1111 |
| n | 0000-0010-0110-1110-1111 |
| o | 0000-0010-0110-1110-1111 |
| p | 0000-0010-0110-0111-1111 |
| q | 0000-0010-0011-1011-1111 |
| r | 0000-0010-0011-0111-1111 |
| s | 0000-0001-1001-1101-1111 |
| t | 0000-0001-1001-1011-1111 |
| u | 0000-0001-0101-1101-1111 |
| v | 0000-0001-0101-0111-1111 |
| w | 0000-0001-0011-1011-1111 |
| x | 0000-0001-0011-0111-1111 |

**Table S5**. Discrete pathways with letter corresponding to the paths in Figure 2.
